# Bis[μ-3,5-bis­(pyridin-2-yl)-1*H*-pyrazole]­bis­[di­bromido­iron(III)]

**DOI:** 10.1107/S1600536813026573

**Published:** 2013-10-02

**Authors:** Nagisa Katsuta, Akio Mishima, Akira Fuyuhiro, Shinya Hayami, Satoshi Kawata

**Affiliations:** a Department of Chemistry, Faculty of Science, Fukuoka University, Nanakuma, Jonan-ku, Fukuoka 814-0180, Japan; b Department of Chemistry, Graduate School of Science, Osaka University, Toyonaka, Osaka 560-0043, Japan; c Department of Chemistry, Graduate School of Science and Technology, Kumamoto University, Kurokami, Kumamoto 860-8555, Japan

## Abstract

The title dinuclear complex, [Fe_2_Br_4_(C_13_H_9_N_4_)_2_], which lies on an inversion center, features two approximately planar bis­(pyridin-2-yl)pyrazole (bpypz^−^) ligands [maximum deviation = 0.082 (3) Å] and four bromide ions. Each Fe^III^ ion is octa­hedrally coordinated by four N atoms of two bpypz^−^ ligands and two Br ions. π–π stacking inter­actions [centroid–centroid distances = 3.7004 (17)–4.0123 (18) Å] are observed between pyridyl and pyrazole rings, and between pyridyl and pyridyl rings of adjacent complex mol­ecules.

## Related literature

For metal complexes of 3,5-bis­(pyridin-2-yl)pyrazole, see: Yoneda, Adachi, Hayami *et al.* (2006 ▶); Yoneda, Adachi, Nishio *et al.* (2006 ▶); Ishikawa *et al.* (2010 ▶); Mishima *et al.* (2011 ▶); Washizaki *et al.* (2012 ▶).
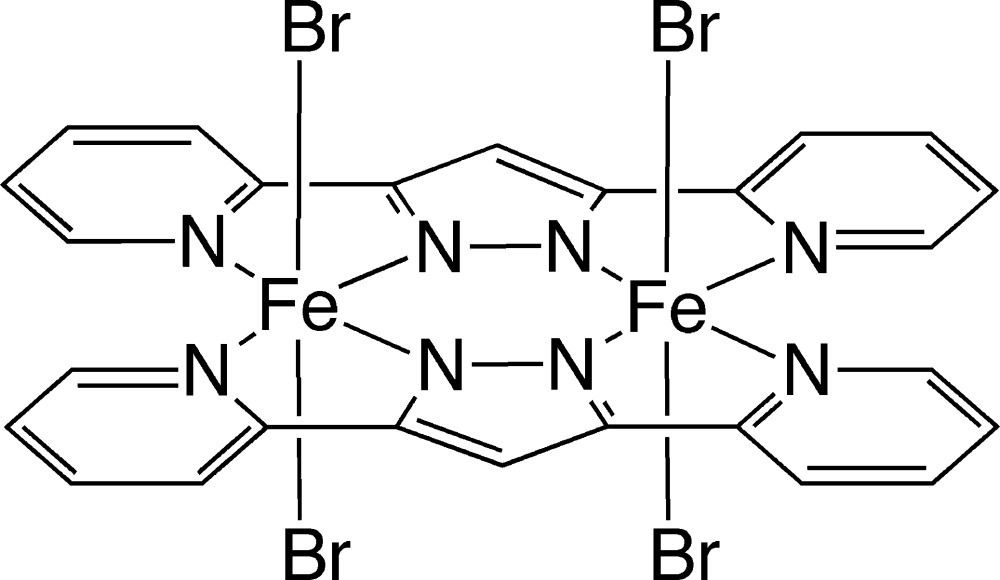


## Experimental

### 

#### Crystal data

[Fe_2_(C_13_H_9_N_4_)_2_Br_4_]
*M* *_r_* = 873.79
Monoclinic, 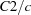

*a* = 18.180 (4) Å
*b* = 14.857 (3) Å
*c* = 10.530 (3) Å
β = 94.646 (3)°
*V* = 2834.7 (10) Å^3^
*Z* = 4
Mo *K*α radiation
μ = 6.71 mm^−1^
*T* = 110 K
0.10 × 0.10 × 0.10 mm

#### Data collection

Rigaku Saturn724 diffractometer
Absorption correction: multi-scan (*REQAB*; Rigaku, 1998 ▶) *T*_min_ = 0.408, *T*_max_ = 0.511
16288 measured reflections
3246 independent reflections
2730 reflections with *I* > 2σ(*I*)
*R*_int_ = 0.031

#### Refinement

*R*[*F* ^2^ > 2σ(*F* ^2^)] = 0.023
*wR*(*F* ^2^) = 0.060
*S* = 1.05
3246 reflections
181 parameters
H-atom parameters constrained
Δρ_max_ = 0.70 e Å^−3^
Δρ_min_ = −0.46 e Å^−3^

### 

Data collection: *CrystalClear* (Rigaku, 2008 ▶); cell refinement: *CrystalClear*; data reduction: *CrystalClear*; program(s) used to solve structure: *SHELXS97* (Sheldrick, 2008 ▶); program(s) used to refine structure: *SHELXL97* (Sheldrick, 2008 ▶); molecular graphics: *CrystalStructure* (Rigaku, 2010 ▶); software used to prepare material for publication: *CrystalStructure*.

## Supplementary Material

Crystal structure: contains datablock(s) General, I. DOI: 10.1107/S1600536813026573/is5304sup1.cif

Structure factors: contains datablock(s) I. DOI: 10.1107/S1600536813026573/is5304Isup2.hkl

Additional supplementary materials:  crystallographic information; 3D view; checkCIF report

## Figures and Tables

**Table 1:** Selected bond lengths (Å)

Br1—Fe1	2.5119 (6)
Br2—Fe1	2.4652 (6)
Fe1—N1	2.1882 (19)
Fe1—N2	2.070 (2)
Fe1—N3^i^	2.0683 (19)
Fe1—N4^i^	2.183 (2)

Symmetry code: (i) 
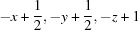
.

**Fig. 1.**
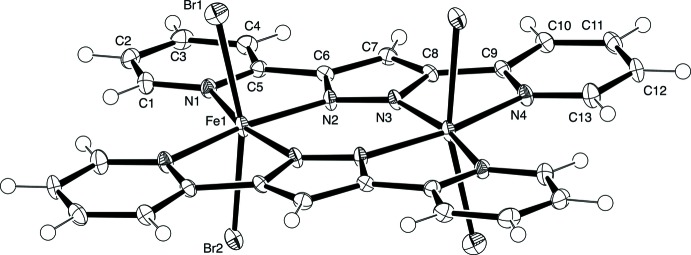
An ORTEP drawing of the title complex, showing 50% probability displacement elipsoids.

**Fig. 2.**
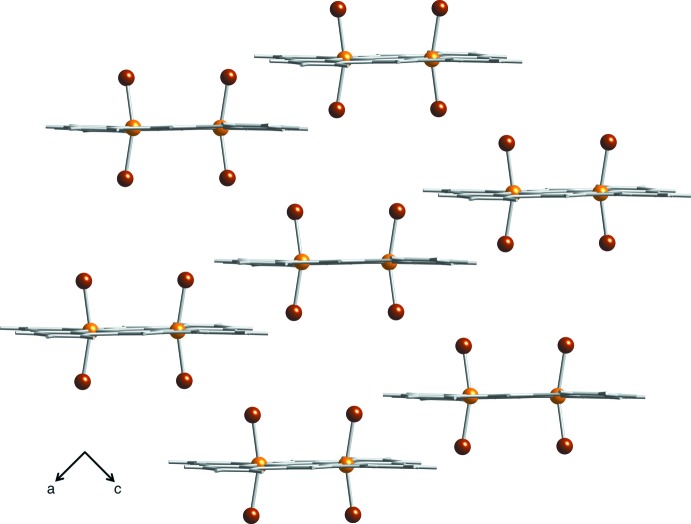
A packing diagram of the title compound, viewed along the b axis.

## supplementary crystallographic information

### 1. Comment

3,5-Bis(pyridin-2-yl)pyrazole[Hbpypz] is a versatile ligand in the construction of a series of mononuclear, dinuclear and polynuclear complexes (Yoneda, Adachi, Hayami *et al.*, 2006; Yoneda, Adachi, Nishio *et al.*, 2006; Ishikawa *et al.*, 2010). The dinuclear complexes show the structure where two bpypz^-^ ions are bridging two metal ions with the axial coordination sites. This kind of dinuclear complexes with transition metal ions were reported previously (Mishima *et al.*, 2011; Washizaki *et al.*, 2012). We have succeeded in synthesizing the title compound that contains iron(III) ion for the first time. To the best of our knowledge, similar compounds with the only iron(II) ions in it (Yoneda, Adachi, Hayami *et al.*, 2006; Yoneda, Adachi, Nishio *et al.*, 2006). In the dinuclear complex, four N donors from two deprotonated tetrahedral bridging bpypz^-^ ligands make an equatorial plane (Table 1). The iron(III) ions are six-coordinated and the axial positions are occupied by bromide ions. From the Mössbauer measurement, the valence of all iron ions is trivalent. There are π–π stacking interactions between pyridyl and pyrazole rings and between pyridyl and pyridyl rings [centroid-centroid distances 3.7004 (17) Å, 4.0123 (18) Å and 4.0022 (18) Å] to form a three-dimensional structure.

### 2. Experimental

A methanolic solution of FeBr_3_ (5 ml, 5 mmol*L*^-1^) was transferred to a glass tube, and methanolic solution of Hbpypz (5 ml, 5 mmol*L*^-1^) was poured into the glass tube without mixing the solutions. Black crystals began to form at ambient temperature within one week (yield 58%). Element analysis: calcd (%) for C_26_H_18_Fe_2_N_8_Br_4_: C 35.74, H 2.08, N 12.83; found; C 35.97, H 2.26, N 12.67.

### 3. Refinement

The C-bound hydrogen atoms in the bpypz^-^ ion were placed at calculated positions (C—H = 0.95 Å) and were treated as riding on their parent atoms with *U*_iso_(H) set to 1.2*U*_eq_(C).

### Crystal data

**Table:**

[Fe_2_(C_13_H_9_N_4_)_2_Br_4_]	*F*(000) = 1688.00
*M**_r_* = 873.79	*D*_x_ = 2.047 Mg m^−^^3^
Monoclinic, *C*2/*c*	Mo *K*α radiation, λ = 0.71075 Å
Hall symbol: -C 2yc	Cell parameters from 3978 reflections
*a* = 18.180 (4) Å	θ = 3.1–27.5°
*b* = 14.857 (3) Å	µ = 6.71 mm^−^^1^
*c* = 10.530 (3) Å	*T* = 110 K
β = 94.646 (3)°	Block, black
*V* = 2834.7 (10) Å^3^	0.10 × 0.10 × 0.10 mm
*Z* = 4	

### Data collection

**Table:**

Rigaku Saturn724 diffractometer	2730 reflections with *F*^2^ > 2σ(*F*^2^)
Detector resolution: 7.111 pixels mm^-1^	*R*_int_ = 0.031
ω scans	θ_max_ = 27.5°
Absorption correction: multi-scan (*REQAB*; Rigaku, 1998)	*h* = −23→23
*T*_min_ = 0.408, *T*_max_ = 0.511	*k* = −19→18
16288 measured reflections	*l* = −13→13
3246 independent reflections	

### Refinement

**Table:**

Refinement on *F*^2^	Secondary atom site location: difference Fourier map
*R*[*F*^2^ > 2σ(*F*^2^)] = 0.023	Hydrogen site location: inferred from neighbouring sites
*wR*(*F*^2^) = 0.060	H-atom parameters constrained
*S* = 1.05	*w* = 1/[σ^2^(*F*_o_^2^) + (0.0344*P*)^2^] where *P* = (*F*_o_^2^ + 2*F*_c_^2^)/3
3246 reflections	(Δ/σ)_max_ = 0.001
181 parameters	Δρ_max_ = 0.70 e Å^−^^3^
0 restraints	Δρ_min_ = −0.46 e Å^−^^3^
Primary atom site location: structure-invariant direct methods	

### Special details

**Table:**

Geometry. ENTER SPECIAL DETAILS OF THE MOLECULAR GEOMETRY
Refinement. Refinement was performed using all reflections. The weighted *R*-factor (*wR*) and goodness of fit (*S*) are based on *F*^2^. *R*-factor (gt) are based on *F*. The threshold expression of *F*^2^ > 2.0 σ(*F*^2^) is used only for calculating *R*-factor (gt).

### Fractional atomic coordinates and isotropic or equivalent isotropic displacement parameters (Å^2^)

**Table:**

	*x*	*y*	*z*	*U*_iso_*/*U*_eq_	
Br1	0.443797 (14)	0.261741 (15)	0.48610 (2)	0.01790 (7)	
Br2	0.229060 (14)	0.271406 (15)	0.16980 (2)	0.01985 (8)	
Fe1	0.323234 (19)	0.26281 (2)	0.35150 (3)	0.01290 (9)	
N1	0.35940 (11)	0.39427 (12)	0.28651 (17)	0.0132 (4)	
N2	0.27110 (11)	0.35166 (12)	0.46615 (17)	0.0127 (4)	
N3	0.22342 (11)	0.34385 (12)	0.55761 (17)	0.0129 (4)	
N4	0.13421 (11)	0.35514 (12)	0.74213 (18)	0.0142 (4)	
C1	0.40369 (13)	0.41136 (16)	0.1936 (3)	0.0170 (5)	
C2	0.42317 (13)	0.49806 (17)	0.1611 (3)	0.0189 (6)	
C3	0.39704 (14)	0.56992 (16)	0.2285 (3)	0.0183 (5)	
C4	0.35166 (13)	0.55314 (16)	0.3250 (3)	0.0162 (5)	
C5	0.33295 (12)	0.46438 (15)	0.3509 (2)	0.0125 (5)	
C6	0.28327 (13)	0.44061 (14)	0.4473 (2)	0.0115 (5)	
C7	0.24257 (12)	0.49193 (15)	0.5276 (2)	0.0133 (5)	
C8	0.20575 (12)	0.42818 (14)	0.5948 (2)	0.0116 (5)	
C9	0.15481 (12)	0.43495 (15)	0.6951 (2)	0.0122 (5)	
C10	0.13052 (13)	0.51621 (15)	0.7420 (3)	0.0149 (5)	
C11	0.08420 (13)	0.51551 (16)	0.8403 (3)	0.0181 (6)	
C12	0.06344 (14)	0.43331 (16)	0.8892 (3)	0.0186 (5)	
C13	0.08932 (14)	0.35518 (16)	0.8374 (3)	0.0186 (6)	
H1	0.4224	0.3622	0.1484	0.0204*	
H2	0.4539	0.5081	0.0936	0.0227*	
H3	0.4102	0.6299	0.2087	0.0220*	
H4	0.3335	0.6013	0.3729	0.0195*	
H5	0.2406	0.5556	0.5347	0.0160*	
H6	0.1455	0.5716	0.7070	0.0179*	
H7	0.0670	0.5703	0.8737	0.0218*	
H8	0.0320	0.4309	0.9569	0.0223*	
H9	0.0748	0.2991	0.8706	0.0224*	

### Atomic displacement parameters (Å^2^)

**Table:**

	*U*^11^	*U*^22^	*U*^33^	*U*^12^	*U*^13^	*U*^23^
Br1	0.01636 (13)	0.01757 (13)	0.02030 (13)	0.00300 (9)	0.00476 (9)	−0.00269 (9)
Br2	0.02435 (15)	0.01322 (13)	0.02169 (14)	−0.00149 (10)	0.00004 (10)	0.00022 (9)
Fe1	0.01458 (18)	0.00915 (17)	0.01610 (17)	−0.00004 (13)	0.00820 (13)	−0.00050 (12)
N1	0.0134 (10)	0.0127 (10)	0.0140 (10)	−0.0010 (8)	0.0045 (8)	0.0016 (8)
N2	0.0145 (10)	0.0101 (9)	0.0147 (10)	0.0001 (8)	0.0079 (8)	−0.0001 (8)
N3	0.0127 (10)	0.0106 (10)	0.0163 (10)	0.0013 (8)	0.0067 (8)	−0.0006 (8)
N4	0.0158 (11)	0.0105 (9)	0.0172 (10)	0.0010 (8)	0.0066 (8)	−0.0020 (8)
C1	0.0157 (13)	0.0196 (13)	0.0164 (12)	−0.0010 (10)	0.0055 (10)	−0.0006 (10)
C2	0.0122 (12)	0.0284 (14)	0.0166 (12)	−0.0046 (11)	0.0033 (10)	0.0067 (11)
C3	0.0185 (13)	0.0155 (12)	0.0210 (13)	−0.0039 (10)	0.0010 (11)	0.0075 (10)
C4	0.0167 (13)	0.0126 (12)	0.0194 (13)	−0.0008 (10)	0.0013 (10)	0.0021 (10)
C5	0.0121 (12)	0.0124 (12)	0.0132 (12)	−0.0008 (9)	0.0011 (9)	0.0021 (9)
C6	0.0102 (12)	0.0097 (11)	0.0144 (11)	−0.0002 (9)	0.0005 (9)	−0.0001 (9)
C7	0.0141 (12)	0.0100 (11)	0.0157 (12)	0.0010 (9)	0.0005 (10)	−0.0003 (9)
C8	0.0105 (12)	0.0095 (11)	0.0148 (11)	0.0016 (9)	0.0022 (9)	−0.0022 (9)
C9	0.0088 (11)	0.0133 (11)	0.0146 (11)	−0.0010 (9)	0.0016 (9)	−0.0010 (9)
C10	0.0141 (12)	0.0127 (11)	0.0182 (12)	−0.0007 (10)	0.0026 (10)	−0.0022 (9)
C11	0.0150 (13)	0.0178 (13)	0.0219 (13)	0.0028 (10)	0.0032 (10)	−0.0070 (10)
C12	0.0159 (13)	0.0223 (13)	0.0189 (12)	0.0017 (10)	0.0092 (10)	−0.0026 (10)
C13	0.0196 (13)	0.0158 (12)	0.0217 (13)	−0.0011 (10)	0.0089 (11)	−0.0008 (10)

### Geometric parameters (Å, º)

**Table:**

Br1—Fe1	2.5119 (6)	C5—C6	1.456 (4)
Br2—Fe1	2.4652 (6)	C6—C7	1.395 (4)
Fe1—N1	2.1882 (19)	C7—C8	1.386 (4)
Fe1—N2	2.070 (2)	C8—C9	1.464 (4)
Fe1—N3^i^	2.0683 (19)	C9—C10	1.390 (4)
Fe1—N4^i^	2.183 (2)	C10—C11	1.387 (4)
N1—C1	1.340 (4)	C11—C12	1.389 (4)
N1—C5	1.352 (3)	C12—C13	1.381 (4)
N2—N3	1.352 (3)	C1—H1	0.950
N2—C6	1.357 (3)	C2—H2	0.950
N3—C8	1.359 (3)	C3—H3	0.950
N4—C9	1.350 (3)	C4—H4	0.950
N4—C13	1.344 (4)	C7—H5	0.950
C1—C2	1.386 (4)	C10—H6	0.950
C2—C3	1.387 (4)	C11—H7	0.950
C3—C4	1.382 (4)	C12—H8	0.950
C4—C5	1.394 (4)	C13—H9	0.950
			
N1···C3	2.778 (3)	Br1···H7^viii^	3.2176
N2···N2^i^	3.211 (3)	Br1···H8^ix^	3.3070
N2···C9	3.555 (3)	Br1···H9^ix^	2.9039
N3···N3^i^	3.221 (3)	Br2···H4^ii^	2.7927
N3···C5	3.552 (3)	Br2···H5^iii^	2.9531
N4···C11	2.779 (3)	Br2···H6^iii^	2.8284
C1···C4	2.731 (4)	N1···H5^iii^	3.3653
C2···C5	2.732 (4)	N2···H3^vi^	3.4557
C4···C7	3.162 (4)	N2···H6^iii^	3.5987
C7···C10	3.181 (4)	N3···H7^iii^	3.5460
C9···C12	2.736 (4)	C1···H1^iv^	3.5269
C10···C13	2.723 (4)	C1···H2^iv^	3.5841
Br2···C4^ii^	3.562 (3)	C1···H4^iii^	3.5173
C1···C7^iii^	3.587 (4)	C1···H5^iii^	3.3217
C2···C2^iv^	3.235 (4)	C2···H2^iv^	3.2792
C2···C3^iv^	3.544 (4)	C2···H5^iii^	3.5654
C2···C5^iii^	3.578 (4)	C3···H2^iv^	3.2970
C2···C6^iii^	3.382 (4)	C4···H2^vi^	3.3814
C2···C7^iii^	3.468 (4)	C5···H2^vi^	3.2600
C3···C2^iv^	3.544 (4)	C6···H2^vi^	3.4358
C3···C6^iii^	3.476 (4)	C6···H6^iii^	3.4177
C3···C7^iii^	3.499 (4)	C7···H7^iii^	3.5827
C4···Br2^v^	3.562 (3)	C8···H7^iii^	3.2916
C4···C8^iii^	3.456 (4)	C10···H8^iii^	3.4589
C5···C2^vi^	3.578 (4)	C11···H5^vi^	3.5274
C6···C2^vi^	3.382 (4)	C11···H7^vii^	3.5058
C6···C3^vi^	3.476 (4)	C11···H8^x^	3.2231
C6···C10^iii^	3.438 (4)	C12···H5^vi^	3.4559
C7···C1^vi^	3.587 (4)	C12···H6^vi^	3.5530
C7···C2^vi^	3.468 (4)	C12···H7^x^	3.5799
C7···C3^vi^	3.499 (4)	C12···H8^x^	3.1884
C7···C10^iii^	3.496 (4)	C13···H5^vi^	3.5645
C7···C11^iii^	3.357 (4)	H1···Br1^iv^	3.2719
C8···C4^vi^	3.456 (4)	H1···C1^iv^	3.5269
C8···C11^iii^	3.436 (4)	H1···H1^iv^	3.3987
C10···C6^vi^	3.438 (4)	H1···H4^iii^	3.2504
C10···C7^vi^	3.496 (4)	H2···C1^iv^	3.5841
C11···C7^vi^	3.357 (4)	H2···C2^iv^	3.2792
C11···C8^vi^	3.436 (4)	H2···C3^iv^	3.2970
C11···C11^vii^	3.471 (4)	H2···C4^iii^	3.3814
C12···C12^vii^	3.580 (4)	H2···C5^iii^	3.2600
Fe1···H1	3.2605	H2···C6^iii^	3.4358
Fe1···H9^i^	3.2336	H2···H2^iv^	3.5717
N1···H2	3.2407	H2···H2^xi^	2.7001
N1···H4	3.2527	H2···H3^iv^	3.5888
N1···H9^i^	3.5694	H2···H4^iii^	3.4658
N2···H5	3.1742	H3···Br1^iii^	2.9480
N3···H5	3.1726	H3···N2^iii^	3.4557
N4···H6	3.2453	H3···H2^iv^	3.5888
N4···H8	3.2424	H3···H3^iv^	3.3113
C1···H3	3.2521	H4···Br2^v^	2.7927
C1···H9^i^	3.2291	H4···C1^vi^	3.5173
C2···H4	3.2519	H4···H1^vi^	3.2504
C3···H1	3.2422	H4···H2^vi^	3.4658
C4···H2	3.2513	H5···Br2^vi^	2.9531
C4···H5	3.1113	H5···N1^vi^	3.3653
C5···H1	3.1713	H5···C1^vi^	3.3217
C5···H3	3.2556	H5···C2^vi^	3.5654
C5···H5	2.9888	H5···C11^iii^	3.5274
C6···H4	2.6951	H5···C12^iii^	3.4559
C7···H4	2.9097	H5···C13^iii^	3.5645
C7···H6	2.9367	H6···Br2^vi^	2.8284
C8···H6	2.7104	H6···N2^vi^	3.5987
C9···H5	2.9872	H6···C6^vi^	3.4177
C9···H7	3.2585	H6···C12^iii^	3.5530
C9···H9	3.1692	H6···H8^iii^	3.2113
C10···H5	3.1329	H7···Br1^xii^	3.2176
C10···H8	3.2546	H7···N3^vi^	3.5460
C11···H9	3.2375	H7···C7^vi^	3.5827
C12···H6	3.2537	H7···C8^vi^	3.2916
C13···H1^i^	3.2409	H7···C11^vii^	3.5058
C13···H7	3.2484	H7···C12^x^	3.5799
H1···H2	2.3264	H7···H7^vii^	3.4188
H1···H9^i^	2.4051	H7···H8^x^	2.6342
H2···H3	2.3513	H8···Br1^xiii^	3.3070
H3···H4	2.3459	H8···C10^vi^	3.4589
H4···H5	2.5858	H8···C11^x^	3.2231
H5···H6	2.6162	H8···C12^x^	3.1884
H6···H7	2.3505	H8···H6^vi^	3.2113
H7···H8	2.3563	H8···H7^x^	2.6342
H8···H9	2.3194	H8···H8^x^	2.5629
Br1···H1^iv^	3.2719	H9···Br1^xiii^	2.9039
Br1···H3^vi^	2.9480	H9···H9^vii^	3.5667
			
Br1—Fe1—Br2	163.258 (18)	C4—C5—C6	122.7 (2)
Br1—Fe1—N1	84.93 (5)	N2—C6—C5	117.1 (2)
Br1—Fe1—N2	95.44 (6)	N2—C6—C7	110.0 (2)
Br1—Fe1—N3^i^	96.08 (6)	C5—C6—C7	132.8 (2)
Br1—Fe1—N4^i^	85.69 (6)	C6—C7—C8	103.8 (2)
Br2—Fe1—N1	85.32 (5)	N3—C8—C7	110.3 (2)
Br2—Fe1—N2	95.60 (6)	N3—C8—C9	116.7 (2)
Br2—Fe1—N3^i^	96.53 (6)	C7—C8—C9	132.9 (2)
Br2—Fe1—N4^i^	86.58 (6)	N4—C9—C8	114.5 (2)
N1—Fe1—N2	77.09 (8)	N4—C9—C10	121.8 (3)
N1—Fe1—N3^i^	166.76 (8)	C8—C9—C10	123.6 (2)
N1—Fe1—N4^i^	116.67 (8)	C9—C10—C11	119.2 (3)
N2—Fe1—N3^i^	89.68 (8)	C10—C11—C12	118.9 (3)
N2—Fe1—N4^i^	166.23 (8)	C11—C12—C13	118.8 (3)
N3^i^—Fe1—N4^i^	76.56 (8)	N4—C13—C12	122.8 (3)
Fe1—N1—C1	127.64 (16)	N1—C1—H1	118.756
Fe1—N1—C5	113.76 (15)	C2—C1—H1	118.757
C1—N1—C5	118.6 (2)	C1—C2—H2	120.548
Fe1—N2—N3	135.33 (14)	C3—C2—H2	120.549
Fe1—N2—C6	116.60 (16)	C2—C3—H3	120.420
N3—N2—C6	108.03 (18)	C4—C3—H3	120.433
Fe1^i^—N3—N2	134.91 (14)	C3—C4—H4	120.531
Fe1^i^—N3—C8	117.24 (16)	C5—C4—H4	120.537
N2—N3—C8	107.84 (18)	C6—C7—H5	128.129
Fe1^i^—N4—C9	114.88 (16)	C8—C7—H5	128.117
Fe1^i^—N4—C13	126.62 (16)	C9—C10—H6	120.378
C9—N4—C13	118.5 (2)	C11—C10—H6	120.397
N1—C1—C2	122.5 (3)	C10—C11—H7	120.565
C1—C2—C3	118.9 (3)	C12—C11—H7	120.574
C2—C3—C4	119.1 (3)	C11—C12—H8	120.618
C3—C4—C5	118.9 (3)	C13—C12—H8	120.606
N1—C5—C4	121.9 (2)	N4—C13—H9	118.594
N1—C5—C6	115.4 (2)	C12—C13—H9	118.589
			
Br1—Fe1—N1—C1	84.08 (14)	Fe1—N2—N3—C8	−177.09 (13)
Br1—Fe1—N1—C5	−95.49 (11)	Fe1—N2—C6—C5	−1.4 (3)
Br1—Fe1—N2—N3	−99.09 (17)	Fe1—N2—C6—C7	177.79 (11)
Br1—Fe1—N2—C6	83.63 (12)	N3—N2—C6—C5	−179.43 (16)
Br1—Fe1—N3^i^—N2^i^	98.43 (16)	N3—N2—C6—C7	−0.2 (3)
Br1—Fe1—N3^i^—C8^i^	−83.02 (12)	C6—N2—N3—Fe1^i^	−178.29 (16)
Br1—Fe1—N4^i^—C9^i^	94.73 (12)	C6—N2—N3—C8	0.4 (2)
Br1—Fe1—N4^i^—C13^i^	−83.63 (14)	Fe1^i^—N3—C8—C7	178.53 (11)
Br2—Fe1—N1—C1	−82.30 (14)	Fe1^i^—N3—C8—C9	−0.5 (3)
Br2—Fe1—N1—C5	98.13 (11)	N2—N3—C8—C7	−0.4 (3)
Br2—Fe1—N2—N3	93.51 (17)	N2—N3—C8—C9	−179.40 (16)
Br2—Fe1—N2—C6	−83.77 (12)	Fe1^i^—N4—C9—C8	−3.6 (3)
Br2—Fe1—N3^i^—N2^i^	−92.60 (16)	Fe1^i^—N4—C9—C10	178.07 (13)
Br2—Fe1—N3^i^—C8^i^	85.95 (12)	Fe1^i^—N4—C13—C12	−178.31 (13)
Br2—Fe1—N4^i^—C9^i^	−100.13 (12)	C9—N4—C13—C12	−0.0 (4)
Br2—Fe1—N4^i^—C13^i^	81.51 (14)	C13—N4—C9—C8	177.94 (18)
N1—Fe1—N2—N3	177.39 (18)	C13—N4—C9—C10	−0.4 (3)
N1—Fe1—N2—C6	0.12 (12)	N1—C1—C2—C3	1.2 (4)
N2—Fe1—N1—C1	−179.14 (16)	C1—C2—C3—C4	−0.8 (4)
N2—Fe1—N1—C5	1.29 (11)	C2—C3—C4—C5	−0.6 (4)
N1—Fe1—N4^i^—C9^i^	176.84 (11)	C3—C4—C5—N1	1.7 (4)
N1—Fe1—N4^i^—C13^i^	−1.51 (18)	C3—C4—C5—C6	−177.58 (19)
N4^i^—Fe1—N1—C1	1.50 (17)	N1—C5—C6—N2	2.6 (3)
N4^i^—Fe1—N1—C5	−178.07 (10)	N1—C5—C6—C7	−176.4 (2)
N2—Fe1—N3^i^—N2^i^	3.00 (17)	C4—C5—C6—N2	−178.08 (19)
N2—Fe1—N3^i^—C8^i^	−178.45 (13)	C4—C5—C6—C7	2.9 (4)
N3^i^—Fe1—N2—N3	−3.02 (17)	N2—C6—C7—C8	−0.0 (3)
N3^i^—Fe1—N2—C6	179.70 (13)	C5—C6—C7—C8	179.0 (3)
N3^i^—Fe1—N4^i^—C9^i^	−2.58 (12)	C6—C7—C8—N3	0.3 (3)
N3^i^—Fe1—N4^i^—C13^i^	179.07 (16)	C6—C7—C8—C9	179.0 (2)
N4^i^—Fe1—N3^i^—N2^i^	−177.49 (18)	N3—C8—C9—N4	2.7 (3)
N4^i^—Fe1—N3^i^—C8^i^	1.06 (12)	N3—C8—C9—C10	−178.92 (17)
Fe1—N1—C1—C2	−179.70 (13)	C7—C8—C9—N4	−176.0 (2)
Fe1—N1—C5—C4	178.28 (13)	C7—C8—C9—C10	2.3 (4)
Fe1—N1—C5—C6	−2.4 (2)	N4—C9—C10—C11	0.4 (4)
C1—N1—C5—C4	−1.3 (3)	C8—C9—C10—C11	−177.79 (18)
C1—N1—C5—C6	177.99 (17)	C9—C10—C11—C12	0.0 (4)
C5—N1—C1—C2	−0.2 (3)	C10—C11—C12—C13	−0.4 (4)
Fe1—N2—N3—Fe1^i^	4.3 (4)	C11—C12—C13—N4	0.4 (4)

Symmetry codes: (i) −*x*+1/2, −*y*+1/2, −*z*+1; (ii) −*x*+1/2, *y*−1/2, −*z*+1/2; (iii) *x*, −*y*+1, *z*−1/2; (iv) −*x*+1, *y*, −*z*+1/2; (v) −*x*+1/2, *y*+1/2, −*z*+1/2; (vi) *x*, −*y*+1, *z*+1/2; (vii) −*x*, *y*, −*z*+3/2; (viii) −*x*+1/2, *y*−1/2, −*z*+3/2; (ix) *x*+1/2, −*y*+1/2, *z*−1/2; (x) −*x*, −*y*+1, −*z*+2; (xi) −*x*+1, −*y*+1, −*z*; (xii) −*x*+1/2, *y*+1/2, −*z*+3/2; (xiii) *x*−1/2, −*y*+1/2, *z*+1/2.
